# Supplementary figures and images for: Health-Related Quality of Life due to malaria in the Brazilian Amazon using EQ-5D-3L
Source: PLoS Negl Trop Dis. 2024 Dec 19;18(12):e0012739. doi: 10.1371/journal.pntd.0012739 (PMC11698565; doi:10.1371/journal.pntd.0012739)

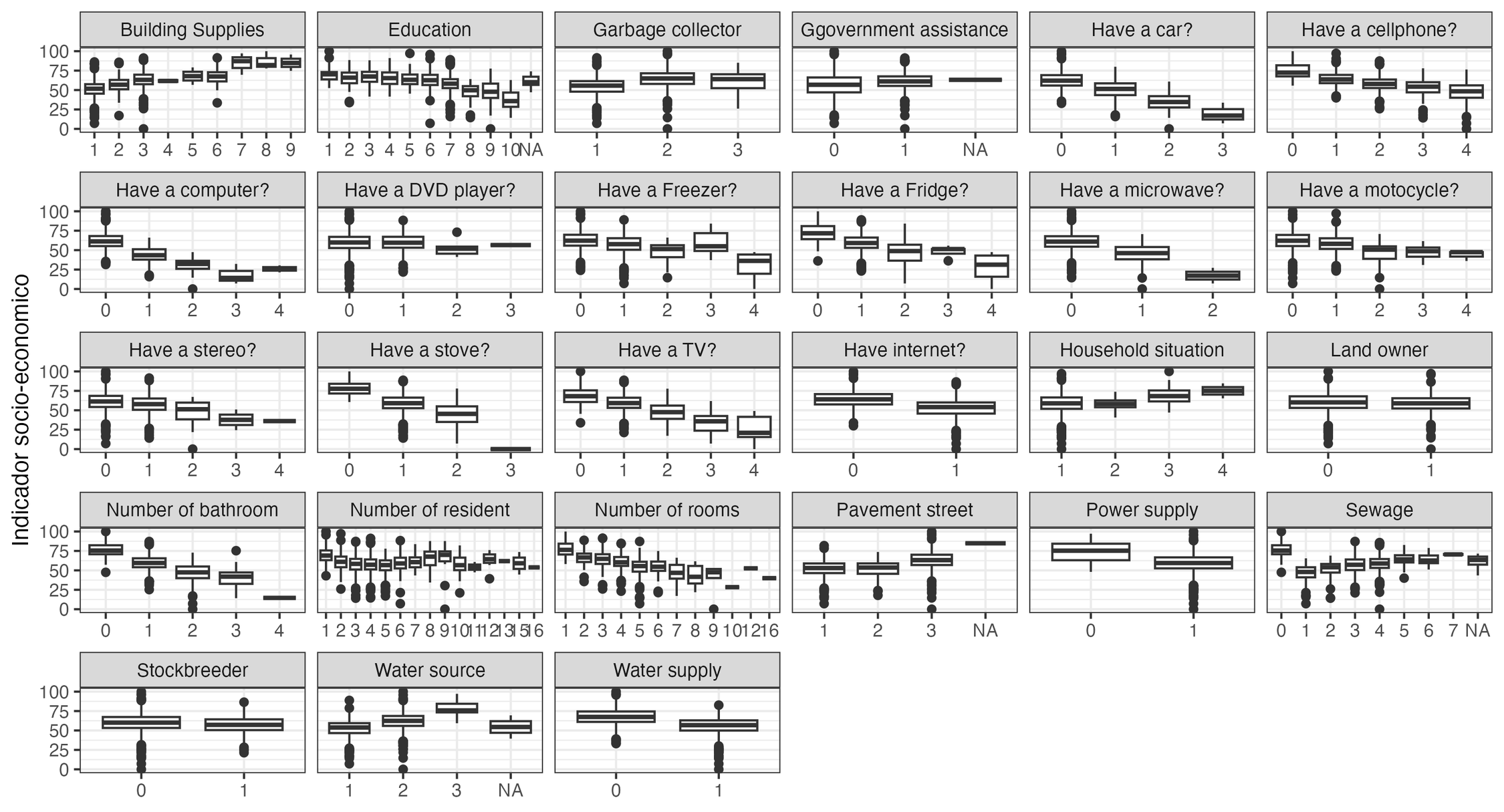

Supplement: S1 Fig — (TIF) [file pntd.0012739.s001.tif]
