# Supplementary material for: Health-Related Quality of Life due to malaria in the Brazilian Amazon using EQ-5D-3L
Source: PLoS Negl Trop Dis. 2024 Dec 19;18(12):e0012739. doi: 10.1371/journal.pntd.0012739 (PMC11698565; doi:10.1371/journal.pntd.0012739)
Supplement: S1 Table — (DOCX) [file pntd.0012739.s002.docx]

| **Table S2.** **Health state of HRQOL malaria patients measured with EQ-5D-3L.** | | | | | | | |
| --- | --- | --- | --- | --- | --- | --- | --- |
| **Control** | | | | **Treatment** | | | |
| **Health State** | **Health utility** | **Frequency** | **Percent** | **Health State** | **Health utility** | **Frequency** | **Percent** |
| 11121 | 0.879 | 180 | 26.51 | 11111 | 1.00 | 82 | 16.40 |
| 11111 | 1000 | 158 | 23.27 | 11121 | 0.88 | 81 | 16.20 |
| 11122 | 0.817 | 93 | 13.70 | 11122 | 0.82 | 41 | 8.20 |
| 11112 | 0.884 | 26 | 3.83 | 21222 | 0.59 | 17 | 3.40 |
| 11131 | 0.746 | 19 | 2.80 | 21122 | 0.69 | 15 | 3.00 |
| 11123 | 0.765 | 17 | 2.50 | 11112 | 0.88 | 14 | 2.80 |
| 21121 | 0.751 | 15 | 2.21 | 11222 | 0.72 | 14 | 2.80 |
| 21122 | 0.689 | 15 | 2.21 | 11131 | 0.75 | 13 | 2.60 |
| 11133 | 0.632 | 10 | 1.47 | 11132 | 0.68 | 13 | 2.60 |
| 22222 | 0.472 | 9 | 1.33 | 21121 | 0.75 | 12 | 2.40 |
| 11113 | 0.832 | 8 | 1.18 | 11123 | 0.77 | 10 | 2.00 |
| 21132 | 0.556 | 7 | 1.03 | 21232 | 0.46 | 9 | 1.80 |
| 21232 | 0.461 | 7 | 1.03 | 22222 | 0.47 | 9 | 1.80 |
| 22221 | 0.534 | 7 | 1.03 | 11133 | 0.63 | 8 | 1.60 |
| 11132 | 0.684 | 6 | 0.88 | 11221 | 0.78 | 8 | 1.60 |
| 11221 | 0.783 | 6 | 0.88 | 21221 | 0.66 | 8 | 1.60 |
| 21221 | 0.655 | 6 | 0.88 | 22233 | 0.29 | 8 | 1.60 |
| 21222 | 0.594 | 6 | 0.88 | 21231 | 0.52 | 7 | 1.40 |
| 11222 | 0.722 | 5 | 0.74 | 21133 | 0.51 | 6 | 1.20 |
| 11223 | 0.670 | 5 | 0.74 | 21233 | 0.41 | 6 | 1.20 |
| 22233 | 0.288 | 5 | 0.74 | 11232 | 0.59 | 5 | 1.00 |
| 21123 | 0.638 | 4 | 0.59 | 21131 | 0.62 | 5 | 1.00 |
| 21231 | 0.522 | 4 | 0.59 | 21132 | 0.56 | 5 | 1.00 |
| 22232 | 0.339 | 4 | 0.59 | 11231 | 0.65 | 4 | 0.80 |
| 21111 | 0.818 | 3 | 0.44 | 11233 | 0.54 | 4 | 0.80 |
| 21131 | 0.618 | 3 | 0.44 | 21123 | 0.64 | 4 | 0.80 |
| 21233 | 0.409 | 3 | 0.44 | 22223 | 0.42 | 4 | 0.80 |
| 22122 | 0.568 | 3 | 0.44 | 22232 | 0.34 | 4 | 0.80 |
| 22211 | 0.601 | 3 | 0.44 | 31221 | 0.38 | 4 | 0.80 |
| 11212 | 0.789 | 2 | 0.29 | 21111 | 0.82 | 3 | 0.60 |
| 11231 | 0.650 | 2 | 0.29 | 22221 | 0.53 | 3 | 0.60 |
| 11232 | 0.589 | 2 | 0.29 | 22333 | 0.18 | 3 | 0.60 |
| 12122 | 0.696 | 2 | 0.29 | 31232 | 0.19 | 3 | 0.60 |
| 21133 | 0.505 | 2 | 0.29 | 31332 | 0.08 | 3 | 0.60 |
| 21331 | 0.413 | 2 | 0.29 | 32332 | -0.05 | 3 | 0.60 |
| 21332 | 0.351 | 2 | 0.29 | 32333 | -0.10 | 3 | 0.60 |
| 22132 | 0.435 | 2 | 0.29 | 33333 | -0.22 | 3 | 0.60 |
| 11233 | 0.537 | 1 | 0.15 | 11211 | 0.85 | 2 | 0.40 |
| 11321 | 0.674 | 1 | 0.15 | 11321 | 0.67 | 2 | 0.40 |
| 11331 | 0.541 | 1 | 0.15 | 21223 | 0.54 | 2 | 0.40 |
| 12111 | 0.825 | 1 | 0.15 | 22231 | 0.40 | 2 | 0.40 |
| 12112 | 0.763 | 1 | 0.15 | 22332 | 0.23 | 2 | 0.40 |
| 12121 | 0.757 | 1 | 0.15 | 23333 | 0.05 | 2 | 0.40 |
| 12123 | 0.644 | 1 | 0.15 | 31133 | 0.23 | 2 | 0.40 |
| 12132 | 0.563 | 1 | 0.15 | 31312 | 0.28 | 2 | 0.40 |
| 12232 | 0.467 | 1 | 0.15 | 31322 | 0.21 | 2 | 0.40 |
| 13321 | 0.427 | 1 | 0.15 | 31323 | 0.16 | 2 | 0.40 |
| 21211 | 0.723 | 1 | 0.15 | 32221 | 0.26 | 2 | 0.40 |
| 21223 | 0.542 | 1 | 0.15 | 32232 | 0.06 | 2 | 0.40 |
| 21322 | 0.484 | 1 | 0.15 | 32233 | 0.01 | 2 | 0.40 |
| 21323 | 0.433 | 1 | 0.15 | 11113 | 0.83 | 1 | 0.20 |
| 22223 | 0.421 | 1 | 0.15 | 11322 | 0.61 | 1 | 0.20 |
| 22231 | 0.401 | 1 | 0.15 | 11323 | 0.56 | 1 | 0.20 |
| 23121 | 0.504 | 1 | 0.15 | 11332 | 0.48 | 1 | 0.20 |
| 23122 | 0.442 | 1 | 0.15 | 11333 | 0.43 | 1 | 0.20 |
| 23333 | 0.053 | 1 | 0.15 | 12111 | 0.83 | 1 | 0.20 |
| 31122 | 0.414 | 1 | 0.15 | 12121 | 0.76 | 1 | 0.20 |
| 31233 | 0.134 | 1 | 0.15 | 12123 | 0.64 | 1 | 0.20 |
| 31322 | 0.209 | 1 | 0.15 | 12222 | 0.60 | 1 | 0.20 |
| 31332 | 0.076 | 1 | 0.15 | 21211 | 0.72 | 1 | 0.20 |
| 32321 | 0.149 | 1 | 0.15 | 21321 | 0.55 | 1 | 0.20 |
| 33311 | 0.091 | 1 | 0.15 | 21322 | 0.48 | 1 | 0.20 |
| 33333 | -0.223 | 1 | 0.15 | 21333 | 0.30 | 1 | 0.20 |
|  |  |  |  | 22111 | 0.70 | 1 | 0.20 |
|  |  |  |  | 22122 | 0.57 | 1 | 0.20 |
|  |  |  |  | 22212 | 0.54 | 1 | 0.20 |
|  |  |  |  | 23121 | 0.50 | 1 | 0.20 |
|  |  |  |  | 23232 | 0.21 | 1 | 0.20 |
|  |  |  |  | 23323 | 0.19 | 1 | 0.20 |
|  |  |  |  | 31121 | 0.48 | 1 | 0.20 |
|  |  |  |  | 31131 | 0.34 | 1 | 0.20 |
|  |  |  |  | 31231 | 0.25 | 1 | 0.20 |
|  |  |  |  | 31233 | 0.13 | 1 | 0.20 |
|  |  |  |  | 31333 | 0.02 | 1 | 0.20 |
|  |  |  |  | 32133 | 0.11 | 1 | 0.20 |
|  |  |  |  | 32331 | 0.02 | 1 | 0.20 |
|  |  |  |  | 33232 | -0.06 | 1 | 0.20 |
|  |  |  |  | 33233 | -0.11 | 1 | 0.20 |
|  |  |  |  | 33322 | -0.04 | 1 | 0.20 |
|  |  |  |  | 33332 | -0.17 | 1 | 0.20 |
