# Supplementary material for: Health-Related Quality of Life due to malaria in the Brazilian Amazon using EQ-5D-3L
Source: PLoS Negl Trop Dis. 2024 Dec 19;18(12):e0012739. doi: 10.1371/journal.pntd.0012739 (PMC11698565; doi:10.1371/journal.pntd.0012739)
Supplement: Attachment — Submitted filename: PNTD-D-24-01046_rev.pdf [file pntd.0012739.s003.pdf]

PLOS Neglected Tropical Diseases

Health-Related Quality of Life due to malaria in the Brazilian Amazon using EQ-5D-3L

--Manuscript Draft--

**Manuscript Number:**	PNTD-D-24-01046
**Full Title:**	Health-Related Quality of Life due to malaria in the Brazilian Amazon using EQ-5D-3L
**Short Title:**	Health-Related Quality of Life due to malaria in the Brazilian Amazon
**Article Type:**	Research Article
**Keywords:**	health-related quality of life; malaria; Brazilian Amazon

Abstract:

Malaria is a mosquito-borne infectious disease caused by protozoa of the genus Plasmodium. Despite of the progress in malaria control in the last decades, malaria remains a major public health problem, contributing to increased morbidity and mortality in tropical and subtropical countries. Among American countries, Bolivia, Venezuela, and Brazil account for 73% of the cases. In Brazil, the majority of malaria cases is concentrated in Amazon region. This study estimated health-related quality of life (HRQoL) losses due to malaria in endemic areas of the Brazilian Amazon using the EQ-5D-3L instrument. We collected data from a convenience sample of 1,179 individuals aged 18 years or older. To measure the HRQoL loss, we matched individuals from the treatment group (with recent malaria) to those from the control group (without recent malaria) using Propensity Score Matching (PSM) and compared the difference in mean health utility between the groups. The results show a significant loss of HRQoL due to malaria. The mean utility was 0.69 and 0.83 for the treatment and control groups, respectively, representing a loss of quality of life of approximately 14% for individuals with recent malaria episodes. These findings underscore the importance of effective malaria prevention and treatment strategies, especially in areas where adverse socioeconomic conditions and a challenging epidemiological context exacerbate the impact of the disease. Continued investment in malaria control programs and improved access to health services are essential to mitigate the negative impact of this disease on the quality of life of affected populations.

absolute loss

opinion not based on data

**Additional Information:**	
**Question**	**Response**
**Government Employee**<br><br>Are you or any of the contributing authors an employee of the United States government?<br><br>Manuscripts authored by one or more US Government employees are not copyrighted, but are licensed under a CC0 Public Domain Dedication, which allows unlimited distribution and reuse of the article for any lawful purpose. This is a legal requirement for US Government employees.<br><br>This will be typeset if the manuscript is accepted for publication.	No - No authors are employees of the U.S. government.
**Financial Disclosure**	Yes

Powered by Editorial Manager® and ProduXion Manager® from Aries Systems Corporation

Enter a financial disclosure statement that describes the sources of funding for the work included in this submission. Review the submission guidelines for detailed requirements. View published research articles from *PLOS Neglected Tropical Diseases* for specific examples.<br><br>This statement is required for submission and **will appear in the published article** if the submission is accepted. Please make sure it is accurate.<br><br>**Funded studies**<br>Enter a statement with the following details:<br>• Initials of the authors who received each award<br>• Grant numbers awarded to each author<br>• The full name of each funder<br>• URL of each funder website<br>• Did the sponsors or funders play any role in the study design, data collection and analysis, decision to publish, or preparation of the manuscript?<br><br>Did you receive funding for this work?	
Please add funding details.<br>as follow-up to "**Financial Disclosure**<br><br>Enter a financial disclosure statement that describes the sources of funding for the work included in this submission. Review the submission guidelines for detailed requirements. View published research articles from *PLOS Neglected Tropical Diseases* for specific examples.<br><br>This statement is required for submission and **will appear in the published article** if the submission is accepted. Please make sure it is accurate.	This study was funded by Bill & Melinda Gates Foundation and Brazilian Ministry of Health through the National Council for Scientific and Technological Development (CNPq) (Process 442842/2019-8), call CNPq/MS-SCTIE-Decit/ Fundação Bill & Melinda Gates Nº 23/2019 –Pesquisas de prevenção, detecção e combate à Malária. Under the terms of the Foundation's grant, we have signed a Creative Commons Attribution 4.0 Generic License to any version of the accepted manuscript that may result from this submission. M.V.A., K.V.M.S.N., and G.R.G. acknowledge CNPq for the research productivity scholarships (processes 309252/2021-0, 303459/2022-0, 317328/2021-2, respectively). K.V.M.S.N. is also supported in part by Minas Gerais Research Funding Foundation FAPEMIG (PPM-00604-17). A.S.M.S. receives resources from CNPq (381587/2018-5). The funders had no role in study design, data collection and analysis, decision to publish, or preparation of the manuscript.

**Funded studies**<br>Enter a statement with the following details:<br>• Initials of the authors who received each award<br>• Grant numbers awarded to each author<br>• The full name of each funder<br>• URL of each funder website<br>• Did the sponsors or funders play any role in the study design, data collection and analysis, decision to publish, or preparation of the manuscript?<br><br>Did you receive funding for this work?"	
Please select the country of your main research funder (please select carefully as in some cases this is used in fee calculation).<br>as follow-up to "**Financial Disclosure**<br><br>Enter a financial disclosure statement that describes the sources of funding for the work included in this submission. Review the submission guidelines for detailed requirements. View published research articles from *PLOS Neglected Tropical Diseases* for specific examples.<br><br>This statement is required for submission and **will appear in the published article** if the submission is accepted. Please make sure it is accurate.<br><br>**Funded studies**<br>Enter a statement with the following details:<br>• Initials of the authors who received each award<br>• Grant numbers awarded to each author<br>• The full name of each funder<br>• URL of each funder website<br>• Did the sponsors or funders play any role in the study design, data collection and analysis, decision to publish, or preparation of the manuscript?<br><br>Did you receive funding for this work?"	BRAZIL - BR
**Competing Interests**	The authors declare no competing interests

On behalf of all authors, disclose any competing interests that could be perceived to bias this work.<br><br>This statement will be typeset if the manuscript is accepted for publication.<br><br>Review the instructions link below and PLOS NTDs' competing interests policy to determine what information must be disclosed at submission.	
**Data Availability**<br><br>Provide a **Data Availability Statement** in the box below. This statement should detail where the data used in this submission can be accessed. This statement will be typeset if the manuscript is accepted for publication.<br><br>Before publication, authors are required to make all data underlying their findings fully available, without restriction. Review our PLOS Data Policy page for detailed information on this policy. Instructions for writing your Data Availability statement can be accessed via the Instructions link below.	The data and code required to reproduce the results in this article are available on GitHub repository (upon acceptance).

Powered by Editorial Manager® and ProduXion Manager® from Aries Systems Corporation

Click here to access/download;Manuscript;Manuscript_v2.docx

Health-Related Quality of Life due to malaria in the Brazilian Amazon using EQ-5D-3L

Health-Related Quality of Life due to Malaria in the Brazilian Amazon

Mônica Viegas Andrade[1,2], Kenya Valeria Micaela de Souza Noronha[1,2], Gilvan Ramalho Guedes[1], Nayara Abreu Julião[1,2], Lucas Resende de Carvalho[1], Aline de Souza[1], Valéria Andrade Silva[1], Andre Soares Motta-Santos[1,2], Henrique Bracarense[1], Cássio Peterka[3], Marcia C. Castro[4*]

[1] Center for Development and Regional Planning, Universidade Federal de Minas Gerais, Belo Horizonte, Minas Gerais, Brazil.

[2] Center for Health Technology Assessment, Universidade Federal de Minas Gerais, Belo Horizonte, Minas Gerais, Brazil.

[3] Ministry of Health, Brasília, DF, Brazil.

[4] Department of Global Health and Population, Harvard T.H. Chan School of Public Health, Boston, MA, USA.

*Corresponding author:

Marcia C. Castro

E-mail: mcastro@hsph.harvard.edu

ORCID: 0000-0003-4606-2795

// Abstract

Malaria is a mosquito-borne infectious disease caused by protozoa of the genus *Plasmodium*. Despite of the progress in malaria control in the last decades, malaria remains a major public health problem, contributing to increased morbidity and mortality in tropical and subtropical countries. Among American countries, Bolivia, Venezuela, and Brazil account for 73% of the cases. In Brazil, the majority of malaria cases is concentrated in Amazon region. This study estimated health-related quality of life (HRQoL) losses due to malaria in endemic areas of the Brazilian Amazon using the EQ-5D-3L instrument. We collected data from a convenience sample of 1,179 individuals aged 18 years or older. To measure the HRQoL loss, we matched individuals from the treatment group (with recent malaria) to those from the control group (without recent malaria) using Propensity Score Matching (PSM) and compared the difference in mean health utility between the groups. The results show a significant loss of HRQoL due to malaria. The mean utility was 0.69 and 0.83 for the treatment and control groups, respectively, representing a loss of quality of life of approximately 14% for individuals with recent malaria episodes. These findings underscore the importance of effective malaria prevention and treatment strategies, especially in areas where adverse socioeconomic conditions and a challenging epidemiological context exacerbate the impact of the disease. Continued investment in malaria control programs and improved access to health services are essential to mitigate the negative impact of this disease on the quality of life of affected populations.

whta is definied as recent?

Author summary

Malaria is an infectious disease transmitted by mosquitoes, primarily affecting tropical and subtropical regions worldwide. Despite significant efforts to control malaria, the rates of illness and death are still important. In the Americas, Brazil, Venezuela, and Bolivia account for more than 50% of the cases. This study focuses on the Brazilian Amazon, where malaria is most prevalent, accounting for 99.5% of all reported cases in the country. We aimed to understand how malaria affects individuals’ health-related quality of life (HRQoL) in these endemic areas. Using the EQ-5D-3L instrument, we collected data from over 1,100 adults in endemic municipalities of the Brazilian Amazon. We compare the EQ-5D-3L utility scores for those who recently had malaria with those who did not by means of Propensity Score Matching. Our findings revealed that individuals who recently had malaria experienced a significant reduction in HRQoL. Specifically, there was a 14% decrease in EQ-5D-3L utility for those affected by recent malaria episodes. These results highlight the urgent need for malaria prevention and treatment programs. Effective strategies and better access to healthcare are key in reducing the disease's impact on individuals' lives, especially in regions with challenging health environments, and poor socioeconomic conditions and infrastructure.

Introduction

Malaria is a mosquito-borne infectious disease caused by protozoa of the genus *Plasmodium*. Significant progress has been made in malaria control since the 1990s, although progress has stalled or even reversed in some countries since the late 2010s [1]. Malaria remains a major public health problem, contributing to increased morbidity and mortality in tropical and subtropical countries. In 2022, an estimated 249 million cases and 608,000 deaths from malaria occurred worldwide. Although 94% of cases occur in Africa, just three countries (Bolivia, Venezuela, and Brazil) account for 73% of the cases in the Americas [2].

The Brazilian Amazon accounts for 99.5% of all reported malaria cases in the country. Despite a 6.6% decrease in cases between 2021 and 2022, dropping from 140,488 to 131,224 cases, preliminary data from the first half of 2023 indicate an 8.7% increase over the previous year. Furthermore, the number of malaria hospitalizations and deaths in the region has been on an upward trend since 2020 [3]. Several factors contribute to the high incidence of malaria cases in the Brazilian Amazon. The region's environmental characteristics, including high temperatures, humidity, dense vegetation, and low elevation, create favorable conditions for the proliferation of vector mosquitoes [4]. The interplay between geoclimatic and socioeconomic factors (i.e., poverty levels and inadequate housing and sanitary conditions) provides minimal or no protection against mosquitoes, thereby increasing the likelihood of infection [5, 6]. It is worth noting that the border areas of the Brazilian Amazon face intense population mobility that is driven by economic opportunities, especially in mining activities, or by humanitarian crises, such as the recent one experienced in Venezuela. These migratory flows increase the risk of imported malaria cases and also contribute to the reintroduction of the disease in regions where it was previously under control [5, 7, 8]. Additionally, the lack of adequate healthcare infrastructure in indigenous lands and hard-to-reach areas (such as illegal mining areas) hinders early diagnosis and effective treatment [8].

P. falciparum and *P. vivax* are the most prevalent *plasmodium* species worldwide. In Brazil, the incidence of *P. vivax* infections is considerably higher than that of *P. falciparum*, representing about 85% of autochthonous cases registered in 2022 [3]. The time elapsed between mosquito bites and the first disease symptoms varies according to the parasite species: at least seven days for *P. falciparum* and 10 to 30 days for *P. vivax*. Mild symptoms are more prevalent for *P. vivax* infections and include fever, headache, and chills, while severe symptoms, such as fatigue, confusion, seizures, and difficulty breathing are more common for *P. falciparum[2]*. These symptoms can lead to important Health-Related Quality of Life (HRQoL) loss mainly due to the recurrent episodes usually associated to malaria.

Studies aiming to measure HRQoL loss due to malaria are scarce. The most usual measurement is DALYs (disability-adjusted life years), which assess the epidemiological burden of malaria[9, 10]. Only a few studies conducted field research to evaluate HRQoL among patients experiencing malaria episodes [11-14]. Most used the EuroQol (EQ-5D) instrument that defines health status in five dimensions (mobility, self-care, usual activities, pain/discomfort, anxiety/depression). There are two versions of EQ-5D instrument, which vary according to the levels of severity. This instrument allows to estimate the Quality-Adjusted Life Years (QALY) considering societal preferences for health status. All studies using EQ-5D found low levels of HRQoL among patients with malaria. However, it is not possible to infer about HRQoL losses due to the absence of control groups.

This study addresses this gap by using the EQ-5D-3L instrument to measure the loss of quality of life-associated with malaria by comparing individuals with and without the disease. To the best of our knowledge, this is the first application of the EQ-5D-3L to individuals with malaria in the Brazilian Amazon region.

Methods

Study design and data collection

Field study was conducted in nine municipalities covering five states in the Brazilian Amazon region using a convenience sample (Fig 1). Municipalities were selected based on disease incidence, as measured by the 2019 Annual Parasite Index (API), and geographic accessibility conditions. Indigenous and gold mining areas were excluded. The selected municipalities are predominantly small, with the exception of Cruzeiro do Sul – with an estimated population of more than 90,000 inhabitants – and relatively underserved areas [15].

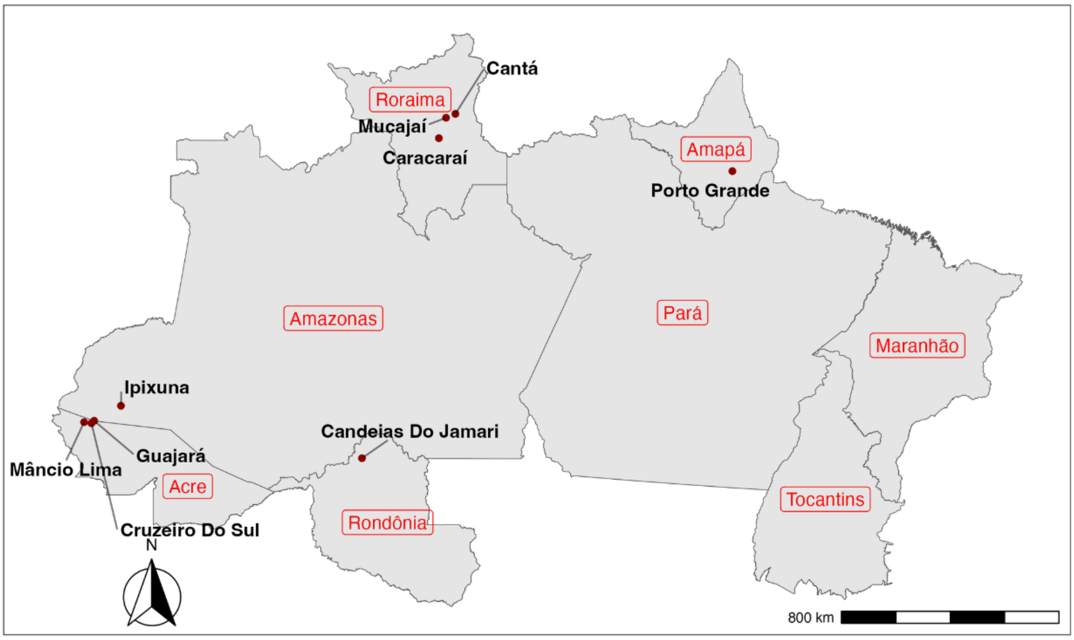

Fig 1. Municipalities of the Brazilian Amazon selected for the study sample.

Note: Maps were created using ggplot package within R environment, version 4.4.0. The base map used is derived from an openly available IBGE shape file source (https://www.ibge.gov.br/geociencias/organizacao-do-territorio/malhas-territoriais.html).

Face-to-face household interviews were conducted in April-May 2022 by independent external research firm. Interviewers were trained and supervised by the study authors, who also

why the effects lasts so long?

conducted quality control of the data collection. The inclusion criterion for households in the study was the presence of at least one resident with a malaria episode between January 2019 and April 2022. Measuring HRQoL losses due to malaria requires a comparison between individuals with malaria (treatment group) and those without the disease (control group). Because it is difficult to find individuals with malaria during the field research, we defined individuals with a recent malaria episode, up to three months before the questionnaire administration, as the treatment group. The control group consisted of individuals without a recent malaria episode. Only one person over the age of 17 was selected from each household. Sample selection was made from a list of eligible residents compiled during the interview, according to pre-established sex and age quotas.

HRQoL was measured by the EQ-5D-3L instrument, developed by the EuroQol Group. It is widely used for measuring HRQoL in adults and validated for use in Brazil [16]. The EQ-5D-3L questionnaire comprises five dimensions (mobility, self-care, usual activities, pain/discomfort, and anxiety/depression), each with three severity levels (1 - no problems, 2 - some problems, and 3 - severe problems). The combination of dimensions/levels generates 243 unique health states. Health states are represented by a sequence of five digits corresponding to the severity level in each dimension. For example, 11111 represents the full health state defined as having no problems in any dimension while 33333 represents the worst health state with extreme problems on all five dimensions. The EQ-5D also includes a visual analog scale (EQ-VAS), where individuals self-assess their health status on a scale ranging from 0 to 100, corresponding to the worst and best imaginable health condition, respectively. In addition to the EQ-5D instrument, interviewers also collected information on sex, age, household characteristics, self-reported general health status, and the presence of pre-existing conditions, including diabetes, hypertension, arthritis, respiratory problems, dengue, yellow fever, and COVID-19.

This study is part of the "The Economic Cost of Malaria in Brazil" grant, funded by the Bill and Melinda Gates Foundation and the National Council for Scientific and Technological Development (CNPq). The project was approved by the Research Ethics Committee of the Federal University of Minas Gerais (Protocol #44774921.1.0000.5149), and the use of the instrument was approved by the EuroQol Group (ID #404310). All participants signed and received a copy of a consent term authorizing their participation in the field survey.

Data analysis

To measure the loss of HRQoL due to malaria, individuals in the treatment group (with recent malaria) were paired with individuals in the control group (without recent malaria) using Propensity Score Matching (PSM), and the difference in mean health utility between the groups was compared. The utility associated with each EQ-5D-3L health state was obtained from the EQ-5D index values estimated by Andrade et al. [17] for the population of Minas Gerais.

PSM is used to minimize sample selection bias in observational studies. The method parametrically assigns each individual in the sample a probability of exposure to the disease – the propensity score (PS) – while controlling for observed characteristics. This creates pairs of individuals from the treatment and control groups with similar propensity scores, and then the average difference between them is subsequently estimated. We estimated the propensity score using a logistic regression model. The explanatory variables include observable characteristics that hypothetically influence the malaria exposure and HRQoL. The characteristics were sex, age, place of residence (urban/rural), socioeconomic status, and dummies for morbidities (diabetes, hypertension, dengue, yellow fever, arthritis, respiratory diseases, and covid). The matching technique used to pair individuals with similar propensity scores was Nearest Neighbor Matching without replacement.

please include reference or be clear: what assets ? school years? why do not use Brazilian Criteria from ABEP?

To assess the socioeconomic status of the participant's h[illegible]onomic index was estimated using Multiple Joint Correspondence Analysis (MCA) according to the presence of household assets. The first principal component explained 49.1% of the variability between households. The indicator was normalized from 0 to 100, corresponding to the lowest and highest socioeconomic levels, respectively.

Frequencies were used to characterize the sample and to analyze the components of the EQ-5D instrument. The Pearson chi-square test was used to compare differences between proportions with a significance level of 95% ($p < 0.05$). Analyses were performed using the R version 4.3.3 statistical software. The packages used for data analysis were tidyverse 2.0.0, MatchIt 4.5.5 for PSM, and ca 0.71_1 for Multiple Joint Correspondence Analysis.

Results

A total of 1,179 individuals were interviewed. Both sexes were equally represented in the sample (49.96% of female and 50.04% of male). Ages ranged from 18 to 88 years, with a mean and a median of 40 years. The age composition indicates a higher share of younger individuals, with 88.8% aged between 18 and 59 years, and 10.2% aged 60 years or older. Men were in the majority at both the base and the top of the age pyramid (Fig 2). The socioeconomic index had a mean score of 49.49 and a standard deviation of 12.5. In terms of self-perceived health, 9.16% rated their health as very good or good, 35.62% as fair, and 40.88% as poor or very poor. Of those surveyed, 97.3% reported having experienced at least one episode of malaria in their lifetime, with a conditional mean of 10.73 episodes.

what does this signify? any external reference?

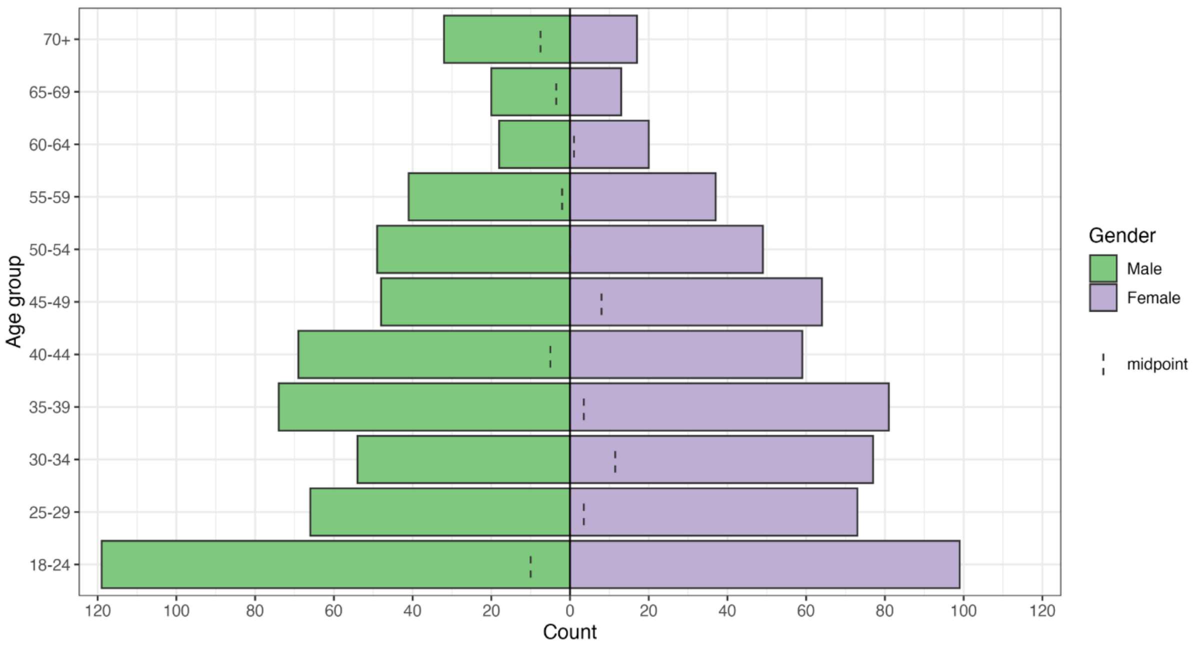

Fig 2. Demographic distribution of the interviewed individual in selected municipalities of the Brazilian Amazon Region, 2022.

Matching results

Of the 1,179 individuals interviewed, 500 were in the treatment group, and 679 were in the control group. The baseline characteristics of individuals in the control and treatment groups are described in Table 1. Before matching, the groups differed in terms of sex (P=0.000), age (P=0.001), presence of arthritis (P=0.002), and respiratory problems (P=0.007). After propensity score matching, the 500 individuals in the treatment group were matched with 500 individuals in the control group, and all observable characteristics were appropriately balanced.

Table 1. Covariate balance in the unmatched and matched samples.

Variables	Before matching			After matching		
	Control	Treatment	P-value	Control	Treatment	P-value
Female	0.545	0.438	<0.001	0.472	0.438	0.281
Age	41.689	38.728	<0.001	39.968	38.728	0.187
Socioeconomic Index	41.092	39.683	0.057	40.249	39.683	0.473
Urban	0.300	0.324	0.390	0.296	0.324	0.339
Covid	0.361	0.354	0.809	0.354	0.354	1.000
Diabetes	0.091	0.082	0.573	0.084	0.082	0.909
Hypertension	0.237	0.218	0.439	0.224	0.218	0.819
Arthritis	0.144	0.088	0.002	0.080	0.088	0.649
Breathing problems	0.135	0.086	0.007	0.080	0.086	0.731
Dengue fever	0.066	0.096	0.068	0.072	0.096	0.731
Yellow fever	0.046	0.026	0.067	0.028	0.026	0.846
Number of observations	679	500		500	500	

Figure 3 shows the density of the estimated propensity scores for the treatment and control groups to check for sufficient overlap between the distributions. The graphs show overlap in all propensity score intervals. It was possible to find a match for every individual in the treatment group within the control group in every interval.

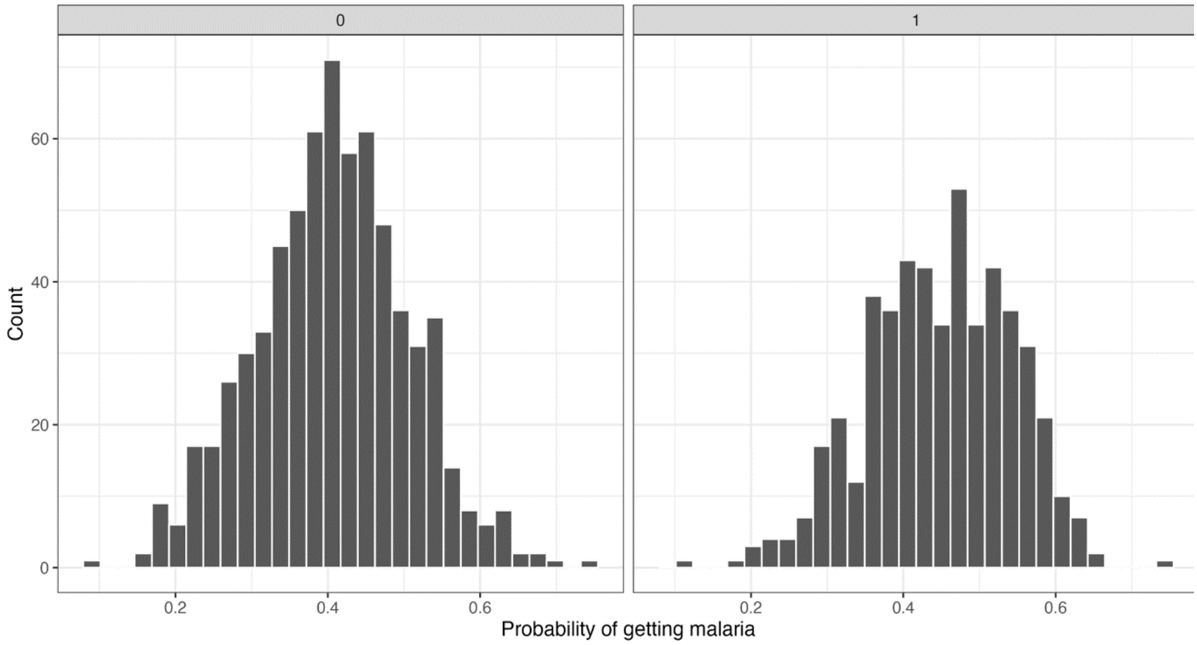

Fig 3. Histograms of the estimated propensity scores by treatment status.

EQ-5D descriptive system and EAV scores

Figure 4 shows the frequencies for each dimension of the EQ-5D-3L descriptive system for individuals in the matched treatment and control groups. Compared to individuals in the treatment group, individuals in the control group reported better health conditions. The percentage of individuals reporting no problems was higher for individuals in the control group in all five health dimensions. Meanwhile, the percentage of individuals reporting moderate or severe problems was higher for individuals in the treatment group in almost all dimensions. "Pain and discomfort" and "anxiety and depression" were the most commonly reported problems for individuals in both groups. In the treatment group, 28% of individuals reported severe problems in the "pain and discomfort" dimension, and 15.8% in the "anxiety and depression" dimension. In the control group, 11.8% of individuals reported severe problems in the "pain and discomfort" dimension, and 8.6% in the "anxiety and depression" dimension. The

health profiles shown in Figure 3 reveal a population already in poor health states, which are further exacerbated by the presence of malaria.

it is not clear how do you separate the effect of malaria with the previous poor condictions - residual counfounding

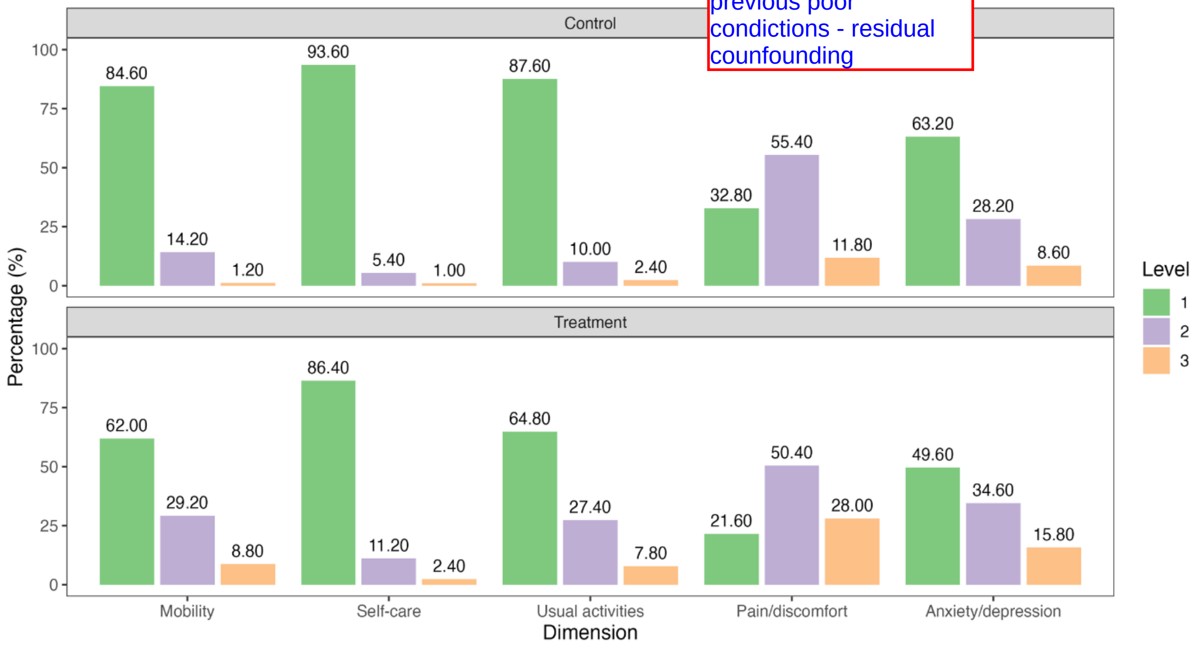

Fig 4. Distribution of individuals according to the EQ-5D-3L dimension and level of severity for treatment and control groups in selected municipalities of the Brazilian Amazon, 2022.

Figure 5 shows the cumulative distribution of health status scores reported by individuals in the treatment and control groups on the EQ-VAS. The score ranges from 0 to 100, representing the worst and best imaginable health status, respectively. The curve for individuals in the treatment group lies above the curve for individuals in the control group, indicating that the frequency of individuals reporting the worst health states is higher for individuals in the treatment group.

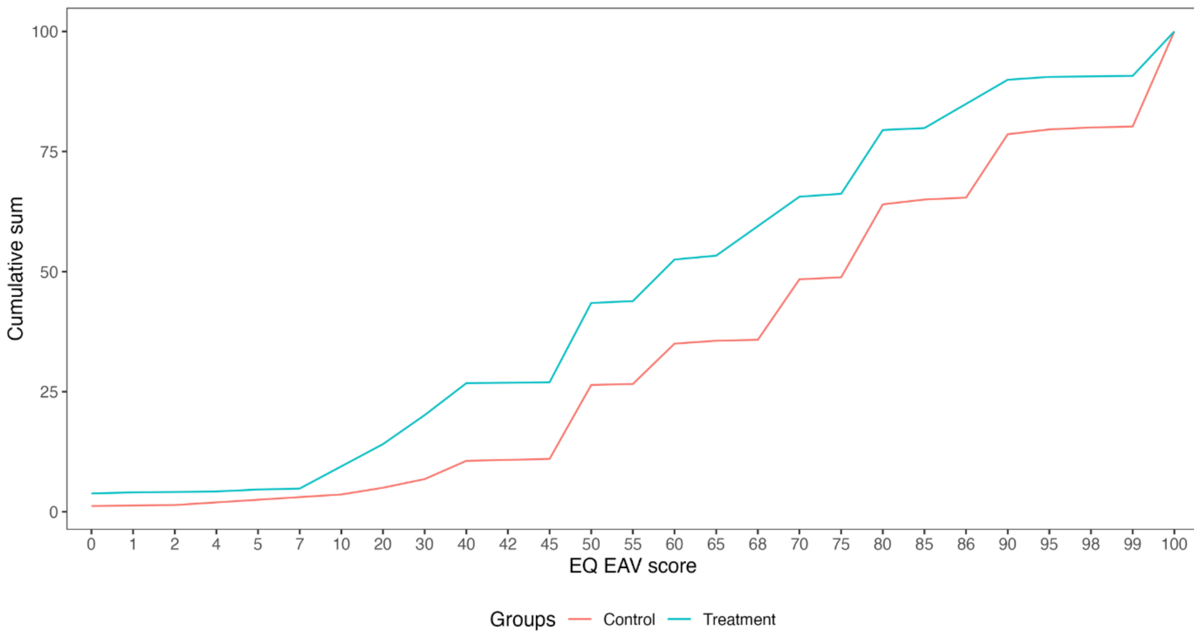

Fig 5. Cumulative distribution of VAS scores for treatment and control groups in selected municipalities of the Brazilian Amazon, 2022.

HRQoL loss due to malaria

The loss of HRQoL due to malaria was estimated by comparing the mean EQ-5D-3L utilities for individuals in the treatment and control groups. A total of 97 health states were identified, with the best health state 11111 occurring 240 times and the worst health state 33333 occurring 4 times (S1 Table). Figure 6 shows the cumulative distribution of health states in descending order of utility for individuals in the treatment and control groups. The mean utility was 0.69 and 0.83 for the treatment and control groups, respectively, representing a loss in HRQoL of approximately 14% for individuals experiencing recent malaria episodes.

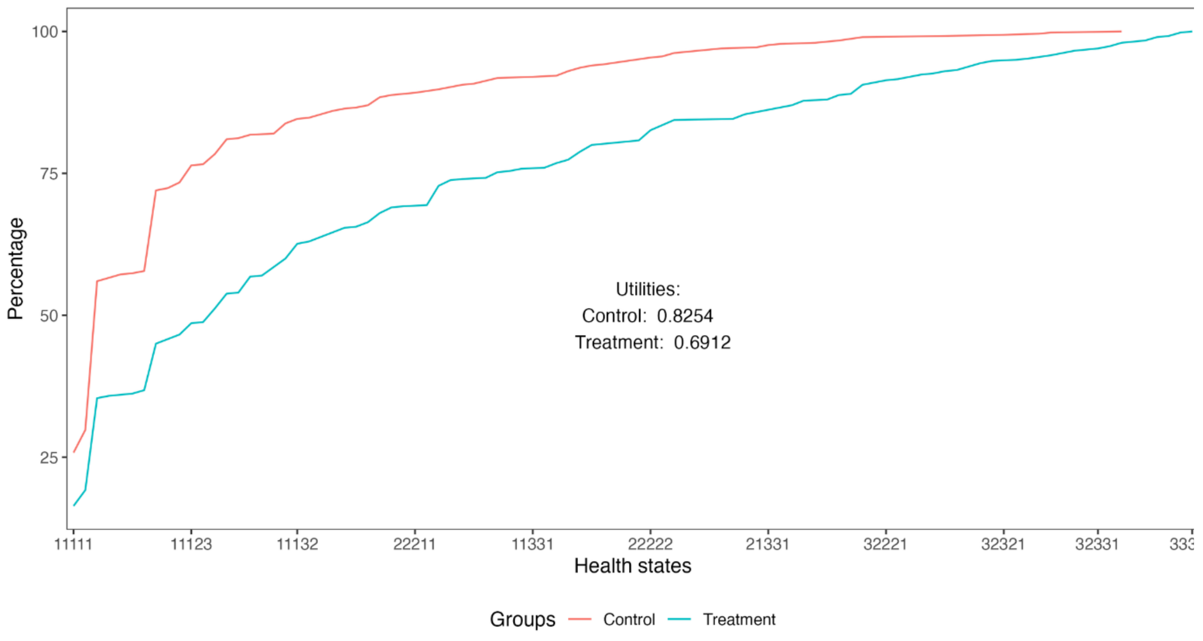

Fig 6. Cumulative distribution of EQ-5D-3L health states for treatment and control groups in selected municipalities of the Brazilian Amazon, 2022.

Discussion

This study assessed the loss of HRQoL due to malaria in the population living in endemic areas of the Brazilian Amazon. A total of 1,179 individuals were interviewed in nine municipalities of five states of the region (Roraima, Amazonas, Amapá, Rondônia, and Acre). The mean EQ-5D-3L score was 0.83 for the control group and 0.69 for the treatment group, indicating a loss of approximately 14% of individuals with recent malaria episodes. In addition, the frequency of individuals in the highest severity levels across all five dimensions of the EQ-5D-3L was higher in the treatment group than in the control group. Pain/discomfort was the most commonly reported dimension, followed by anxiety/depression and usual activities. This score is comparable to the EQ-5D-5L scores associated with severe chronic illness. A meta-analysis using a fixed-effects model suggests that HRQoL for malaria episode is similar to that

for multiple sclerosis (0.67) and lower than that for chronic kidney disease, chronic obstructive pulmonary disease, and cardiovascular disease (0.76) [18].

HRQoL measures overall health and captures different aspects of an individuals' physical and mental health. Both generic and disease-specific instruments can be used to estimate HRQoL. Generic instruments are typically applied to different populations and health conditions and cover broader dimensions such as physical health, emotional well-being, and social aspects. Disease-specific instruments assess quality of life related to specific interventions, conditions, or groups of patients. They typically focus on specific issues related to a condition, such as symptoms and functional limitations. This study used the generic EQ-5D-3L instrument, developed by the EuroQol Group. EQ-5D-3L is one of the most widely used instruments in cost-effectiveness analysis and it is officially recommended by the National Institute for Health and Care Excellence [19]. It is widely used because of its simplicity, rapid administration, and broad applicability, allowing comparisons with other groups and interventions. In Brazil, the Ministry of Health guidelines recommend the use of EQ-5D-3L in economic evaluation studies [20]. Since 2013, the EQ-5D social preferences value set has been available for Brazil [16, 17].

this reference is incomplete

this refernce is incomplete

Previous studies using the EQ-5D have also found lower levels of HRQoL in patients with malaria episodes, with the most affected domains being pain/discomfort and usual activities. Makatita et al. [12] applied the EQ-5D-5L to a sample of 110 malaria patients from Primary Health Care Centers in Indonesia, finding a mean score of 0.49 for all patients. Scores ranged from 0.349 for those with severe malaria to 0.571 for mild cases. Kayiba et al. [13] used the EQ-5D-3L to estimate the economic burden of uncomplicated malaria in the Democratic Republic of Congo, surveying 1,080 patients and finding a mean EQ-5D index score of 0.62, with the majority reporting moderate or severe problems. Jimam et al. [11] assessed the validity of the EQ-5D-5L to measure HRQoL in cases of uncomplicated malaria in Nigeria and reported

good instrument performance with a mean utility score of 0.74. A limitation of these three studies is the lack of control groups, making it difficult to infer HRQoL losses specifically attributable to malaria. To the best of our knowledge this paper is the first to estimate HRQoL losses for a population that has experienced malaria episodes.

The mean EQ-5D score found for the control group in this study (0.83) is lower than previous findings for Brazil: 0.89 for the urban population of Minas Gerais [17] and 0.88 for the metropolitan region of Manaus [21]. In this study, the sampled municipalities are located in endemic areas of the Brazilian Amazon Region, typically characterized by higher socioeconomic vulnerability, difficulty in accessing healthcare services, and poor sanitary conditions. In this context, malaria episodes can exacerbate the vulnerability of health status, in addition to other infectious diseases that are still common in the region, such as yellow fever, hepatitis, dengue, leishmaniasis, leprosy, tuberculosis [22], and intestinal infections [23]. Of those surveyed in this study, 8% reported having had a dengue episode in the previous 12 months, and approximately 4% reported having had yellow fever.

Most respondents had experienced at least one episode of malaria in their lifetime (97.3% of respondents), with 90.24% reporting multiple episodes, yielding a conditional mean of 10.73. These data underscore the high prevalence of malaria reinfection in the region. Personal prevention measures, such as mosquito nets, repellents, and clothing that protects the arms and legs, can significantly reduce the likelihood of contact with vector mosquitoes [24, 25]. Furthermore, medication adherence reduces the risk of complications and reinfection [26]. However, there is evidence of low individual adherence to preventive measures, such as the use of long-lasting insecticidal nets and interrupting treatment when symptoms improve [26-28]. Adherence to antimalarial treatment is associated with socioeconomic conditions, access to healthcare (access to self-medication, monitoring of treatment by healthcare agents), knowledge of the disease, and drug administration - treatment duration, presence of symptoms

I suggest to include the distance from healthcare units sometimes days

it is not prevention, please rewrite the sentence

to treatment or prevention ?

and side effects) [29]. Empirical evidence for the Brazilian Amazon has shown that non-adherence varies from 30 to 40%, depending on the drug treatment and the method used to measure adherence [30].

The main limitation of this study is the sample design. The field survey was conducted using a convenience sample where municipalities were selected based on the Annual Parasite Index (API) and accessibility conditions. Illegal mining areas and indigenous communities were not included in the sample for security and logistical reasons. As these areas are characterized by the higher levels of malaria incidence and poor socioeconomic conditions and access to healthcare, the HRQoL due to malaria may be underestimated. Another limitation is the retrospective assessment of health status experienced during the last malaria episode. However, as a relatively short interval for recent malaria episodes was considered (up to three months before questionnaire administration), recall effects may be minimized. Furthermore, the high prevalence and recurrence of malaria in the Amazon region may influence individuals' perceptions of disease risk. Frequent exposure to malaria may lead to familiarity with the disease, resulting in an underestimation of its impact on quality of life.

Conclusion

To the best of our knowledge, this is the first study to measure malaria-related quality of life loss among the population in endemic areas of the Amazon region. The results show a significant loss of HRQoL due to malaria. These findings underscore the importance of effective malaria prevention and treatment strategies, especially in areas such as the Amazon, where adverse socioeconomic conditions and a challenging epidemiological context exacerbate the impact of the disease. Continued investment in malaria control programs and improved access to health services are essential to mitigate the negative impact of this disease on the quality of life of affected populations.

I suggest to exclude. it is correct, but not based on your data

References

1. World Health Organization. World malaria report 2018. Geneva: World Health Organization; 2018.
2. World malaria report 2023. Geneva: World Health Organization; 2023.
3. Dia da Malária nas Américas - um panorama da malária no Brasil em 2022 e no primeiro semestre de 2023. Brasília; 2024.
4. Daza WG, Muylaert RL, Sobral-Souza T, Landeiro VL. Malaria Risk Drivers in the Brazilian Amazon: Land Use—Land Cover Interactions and Biological Diversity. International Journal of Environmental Research and Public Health. 2023:6497.
5. Ueno TMRL, Lima LNGC, Sardinha DM, Rodrigues YC, Souza HUSd, Teixeira PR, et al. Socio-epidemiological features and spatial distribution of malaria in an area under mining activity in the Brazilian Amazon region. International Journal of Environmental Research and Public Health. 2021:10384.
6. de Oliveira Padilha MA, de Oliveira Melo J, Romano G, de Lima MVM, Alonso WJ, Sallum MAM, Laporta GZ. Comparison of malaria incidence rates and socioeconomic-environmental factors between the states of Acre and Rondônia: a spatio-temporal modelling study. Malaria Journal. 2019:1-13.
7. Arisco NJ, Peterka C, Castro MC. Cross-border malaria in Northern Brazil. Malaria Journal. 2021:1-13.
8. de Aguiar Barros J, Granja F, Pequeno P, Marchesini P, Ferreira da Cruz MdF. Gold miners augment malaria transmission in indigenous territories of Roraima state, Brazil. Malaria Journal. 2022:358.
9. Bezerra JMT, Barbosa DS, Martins-Melo FR, Werneck GL, Braga ÉM, Tauil PL, Carneiro M. Changes in malaria patterns in Brazil over 28 years (1990--2017): results from the Global Burden of Disease Study 2017. Population Health Metrics. 2020:1-15.
10. Kyu HH, Abate D, Abate KH, Abay SM, Abbafati C, Abbasi N, et al. Global, regional, and national disability-adjusted life-years (DALYs) for 359 diseases and injuries and healthy life expectancy (HALE) for 195 countries and territories, 1990--2017: a systematic analysis for the Global Burden of Disease Study 2017. The Lancet. 2018:1859-922.
11. Jimam NS, Ismail NE, Dayom WD. Evaluation of Psychometric Quality of EQ-5D-5L Scale for Assessing Health-Related Quality of Life of Malaria Patients. Value in health regional issues. 2020:15-22.
12. Makatita DA, Nur Hasanah Haris R, Endarti d, Wiedyaningsih C, Andayani Tm. Measurement of Health Related Quality of Life in Malaria Patients in Indonesia using EQ-5D-5L. Journal of Clinical \& Diagnostic Research. 2019.
13. Kayiba NK, Yobi DM, Devleesschauwer B, Mvumbi DM, Kabututu PZ, Likwela JL, et al. Care-seeking behaviour and socio-economic burden associated with uncomplicated malaria in the Democratic Republic of Congo. Malaria journal. 2021:260.
14. Van Damme-Ostapowicz K, Krajewska-Kułak E, Rozwadowska E, Nahorski WL, Olszański R. Quality of life and satisfaction with life of malaria patients in context of acceptance of the disease: quantitative studies. Malaria Journal. 2012:1-10.
15. de IB, Geografia e Estatística. Cidades@. 2024.
16. Santos M, Cintra MA, Monteiro AL, Santos B, Gusmao-Filho FaA, Mônica Viegas, Noronha K, et al. Brazilian valuation of EQ-5D-3L health states: results from a saturation study. 2016:253-63.
17. Andrade MV, Noronha K, Kind P, Maia AC, de Menezes RM, Reis CDB, et al. Societal preferences for EQ-5D health states from a Brazilian population survey. Value in health regional issues. 2013:405-12.

18. Zhou T, Guan H, Wang L, Zhang Y, Rui M, Ma A. Health-related quality of life in patients with different diseases measured with the EQ-5D-5L: a systematic review. Frontiers in Public Health. 2021:675523.
19. Position statement on use of the EQ-5D-5L value set for England (updated October 2019). 2024.
20. Brasil. Diretrizes Metodológicas: Qualidade de Vida em Análises Econômicas. 2023.
21. Silva MT, Caicedo Roa M, Galvao TF. Health-related quality of life in the Brazilian Amazon: a population-based cross-sectional study. Health and quality of life outcomes. 2017:1-9.
22. Aguilar HM, Abad-Franch F, Dias JCP, Junqueira ACV, Coura JR. Chagas disease in the Amazon Region. Memórias do Instituto Oswaldo Cruz. 2007:47-56.
23. Vasconcelos MPA, Sanchez-Arcila JC, Peres L, de Sousa PSF, dos Santos Alvarenga MA, Castro-Alves J, et al. Malarial and intestinal parasitic co-infections in indigenous populations of the Brazilian Amazon rainforest. Journal of Infection and Public Health. 2023:603-10.
24. Vezenegho SB, Adde A, Santi VPd, Issaly J, Carinci R, Gaborit P, et al. High malaria transmission in a forested malaria focus in French Guiana: How can exophagic Anopheles darlingi thwart vector control and prevention measures? Memórias do Instituto Oswaldo Cruz. 2016:561-9.
25. da Silva Ferreira Lima AC, Galardo AKR, Müller JN, de Andrade Corrêa APS, Ribeiro KAN, Silveira GA, et al. Evaluation of Long-lasting insecticidal nets (LLINs) for malaria control in an endemic area in Brazil. Parasites & Vectors. 2023:162.
26. Dinelly KMO, Vitor-Silva S, Brito-Sousa JD, Sampaio VS, Silva MGO, Siqueira AM, et al. Evaluation of the effect of supervised anti-malarial treatment on recurrences of Plasmodium vivax malaria. Malaria journal. 2021:1-6.
27. Sousa JdO, de Albuquerque BC, Coura JR, Suárez-Mutis MC. Use and retention of long-lasting insecticidal nets (LLINs) in a malaria risk area in the Brazilian Amazon: a 5-year follow-up intervention. Malaria journal. 2019:1-13.
28. Almeida ED, Vieira JLF. Factors associated with non-adherence to the treatment of vivax malaria in a rural community from the Brazilian Amazon Basin. Revista da Sociedade Brasileira de Medicina Tropical. 2016:248-51.
29. Pereira EA, Ishikawa EA, Fontes CJ. Adherence to Plasmodium vivax malaria treatment in the Brazilian Amazon Region. Malaria journal. 2011:1-6.
30. Santos HFP, Guaraldo L, Pedro RS, Damasceno LS, Daniel-Ribeiro CT, Brasil P. Methods to assess adult and adolescent patients' adherence to antimalarial treatment: A systematic review. Frontiers in Pharmacology. 2022:796027.

Supporting information

S1 Table. Health state of HRQOL malaria patients measured with EQ-5D-3L.

Click here to access/download
Supporting Information
S1_Table.docx
