# Supplementary material for: Health-Related Quality of Life due to malaria in the Brazilian Amazon using EQ-5D-3L
Source: PLoS Negl Trop Dis. 2024 Dec 19;18(12):e0012739. doi: 10.1371/journal.pntd.0012739 (PMC11698565; doi:10.1371/journal.pntd.0012739)
Supplement: Attachment — Submitted filename: Reviewer Comments.docx [file pntd.0012739.s004.docx]

**Reviewer Comments:**

**Reviewer 1:**

1. The use of DALY-based estimates of malarial disease burden is mentioned (p5-95). If this is relevant at this point in the text, then it needs some clarification as to the different approach taken with QALY estimates. From this reviewer’s perspective, it might be better located as a point for the discussion.

**Answer:** *Thanks for the comment. We agree with reviewer that DALY-based estimates approach is different from QALY estimates. Therefore, we deleted this statement from the manuscript (****page 5****).*

2. The existence of a 2nd version of EQ-5D is not really relevant at this point and could well be removed (p5-99).

**Answer:** *Suggestion accepted. We removed the sentence about the two versions of EQ5D on* ***page 5***

3. (p5-104) EQ-5D is recognised generally as a measure of health-related quality of life (HrQoL) and this term is used later in the manuscript.

**Answer:** *Suggestion accepted. We rewrote the sentence to include the term health-related quality of life (HrQoL)* ***on page 5****.*

*“This study addresses this gap by using the EQ-5D-3L instrument to measure the loss of health-related quality of life (HrQoL) associated with malaria by comparing individuals with and without the disease.”*

4. It is tempting to report mean episodes, but it is hard to understand what is meant by 0.73 of an episode (p9-188). Would it be more meaningful to report median or mode as the indicator of central tendency?

**Answer:** *Suggestion accepted. We rounded the mean to report it as an integer (mean = 11) and added the median and mode* ***on page 10****.*

*“Of those surveyed, 97.3% reported having experienced at least one episode of malaria in their lifetime, with a conditional mean of approximately 11 episodes (median = 6 and mode = 2).”*

5. Table 1 reports sample characteristics, but could benefit from improved labelling, means and percentages? Also, since the treatment group remains unchanged, perhaps the presentation could be rejigged to remove the duplicate?

**Answer:** *Suggestion accepted. We removed the duplicate column for the treatment group and changed the table title to "Sample characteristics and covariate balance in unmatched and matched samples (means reported)"* ***on page 11****.*

6. Figure 3 requires a little attention. The 0 / 1 heading is the indicator of group but this needs a text label. Although the x-axis refers to probabilities, the y-axis is labelled “count”.

**Answer:** *Suggestion accepted. We updated the figure* ***on page 12*** *to include the correct legend, replacing 0 with “control” and 1 with “treatment.” Although this representation of the axes is not the most common, it is appropriate for illustrating the area of common support. In the graph, the x-axis represents the probability of contracting malaria, while the y-axis shows the number of individuals within each probability range. Therefore, the graph helps visualize the distribution of malaria probabilities across the control and treatment groups, highlighting the overlap between the two groups.*

7. The statement regarding overlapping propensity score intervals (p11-205) is difficulty to understand from this graphic.

**Answer:** *Suggestion accepted. We swapped the positions of the graphs in Figure 3* ***on page 12*** *to improve the visualization of the common support area.*

8. EAV (p12-208) appears as a section heading, but also in Figure 5. Needs to read VAS?

**Answer:** *We changed EAV to VAS in the subtitle* ***on page 13*** *and in the title of Figure 5*.

9. Figure 3 (p13) is actually Figure 4?

**Answer:** *The reviewer is right. It was corrected* ***on page 14****.*

*The health profiles shown in Figure 4 reveal a population already in poor health states, which are further exacerbated by the presence of malaria.*

10. Figure 5 y-axis is cumulative percentage? Would it be clearer to show the distribution along the x-axis and the y-axis to indicate mean EQ-VAS. In any case it would be good to have some gridlines as from inspection it seems that 50% of treatment group report a mean EQ VAS of ~50 whereas the control group is ~70 (almost 50% higher). These data could be compared in the Discussion with the preference-weighted HrQoL scores?

**Answer:** *The figure was updated by adding the label “cumulative percentage” on the y-axis and the gridlines. We also incorporated the HRQoL results using EQ-VAS values. The following sentences were included in the results and discussion section.*

*Results section,* ***page 16***

*The HRQoL loss measured using EQ-VAS values was quite similar, 17.5% (EQ-VAS value for control group equal to 71.8 versus 59.3 for treatment group).*

*Discussion section,* ***page 17****.*

*The findings using EQ-VAS values corroborate the EQ-5D-3L results, reflecting a similar magnitude of loss.*

11. Please review the repeated reference to “in selected municipalities of the Brazilian Amazon, 2022” in Figure labels.

**Answer:** *Suggestion accepted. We removed the reference “in selected municipalities of the Brazilian Amazon, 2022” from the figure labels.*

12. The Discussion section includes EQ-5D values from studies in other countries, some based on the 5L version. The two versions are based on difference classification/valuation systems, and this distinction must be made here.

**Answer:** *Suggestion accepted.* *The following sentence was rewritten to clarify this distinction*.

**Page 18**: Although not directly comparable, this score is consistent with the EQ-5D-5L scores associated with severe chronic illness. A meta-analysis using a fixed-effects model suggests that HRQoL for malaria episode is similar to that for multiple sclerosis (0.67) and lower than that for chronic kidney disease, chronic obstructive pulmonary disease, and cardiovascular disease (0.76) [18]

13. This study reports the use of the 3L version of EQ-5D in a convenience sample of selected Brazilian municipalities. The study design is straightforward and the use of propensity matching to achieve a balanced control/treatment sample seems perfectly reasonable.

**13A.** The results are interesting in that they clearly demonstrate differences between respondents reporting a recent malarial episode and those with a more distal experience (if at all). It would have been interesting to know a little more about the recency of any malaria and/or any residual after-effects in the control group. However, given the results it is fair to conclude (as the authors do) that if anything, the study likely underestimates the true picture.

**Answer:** *Suggestion accept.* *In this study, we defined individuals with a recent malaria episode, up to three months before the questionnaire administration (between January and April 2022), as the treatment group. The control group consisted of individuals without a recent malaria episode. To shed some light on the possible residual malaria after-effect, we analyzed the distribution of time elapsed since the last malaria episode among individuals in the control group, relative to January 2022. For the individuals in the control group, the mean time since the last episode was 1.87 years, with a median of 0.91 years. Approximately 70% of these individuals had their most recent episode more than six months before January 2022. This distribution suggests that the health impacts of malaria may have already dissipated by the time of the survey. We added this information in the results section of the new version of the manuscript as following:*

***Page 10****.* Of the 1,179 individuals interviewed, 500 were in the treatment group, and 679 were in the control group. The baseline characteristics of individuals in the control and treatment groups are described in Table 1. Before matching, the groups differed in terms of sex (P=0.000), age (P=0.001), presence of arthritis (P=0.002), and respiratory problems (P=0.007). After propensity score matching, the 500 individuals in the treatment group were matched with 500 individuals in the control group, and all observable characteristics were appropriately balanced. *Among individuals of the control group, the mean time since the last malaria episode was 1.87 years, with a median of 0.91 years; and approximately 70% of them had their most recent episode more than six months before January 2022.*

**13B.** It would have been interesting also, to see further use of the EQ-5D data, perhaps using the EQ-VAS as the dependent variable to examine covariation of morbidity / study group – for example, was there a larger difference in VAS for respondents with/without a COVID history and was that difference amplified in the control/treatment groups?

**Answer**: *This is an intriguing idea for future research. In the current paper, the PSM estimation includes a set of morbidity variables (e.g., diabetes, hypertension, dengue, yellow fever, arthritis, respiratory diseases, and COVID) as independent variables. As a result, the observable morbidity characteristics between the control and treatment groups are already accounted for in the matching process.*

**13C.** More specifically, is there not an obvious further use of the HrQoL results, could they be used to show mean EQ-5D index values by age/sex for the 2 groups? Of course there are limits, but surely it is then a small step to applying those reference values to the municipalities / Region to indicate the potential QALY loss in a single year – even as an order of magnitude? Furthermore, if you monetarise the QALY loss using the World Bank per capita GDP estimate, then this could produce an interesting (even headline-grabbing) result!

**Answer:** *The reviewer raised important points regarding the need for further interpretation of our results. However, the design of our sample does not allow the analysis to be disaggregate by specific demographic groups or at the municipal level. The monetization of QALY losses will be addressed in a future publication from this project, which estimates the economic burden of malaria from a societal perspective. Therefore, we have opted not to include the QALY losses monetization in the present manuscript.*

**Reviewer 2:**

1. You definied 3 months as "recent malaria". please explain why it lasts so long.

**Answer:** *We defined “recent malaria” as cases within the last three months to ensure an adequate treatment group during data collection. The interviewed municipalities had been experiencing a steady decline in malaria cases, and since data collection lasted only one month, there were not enough individuals with active malaria at the time of the interviews. Extending the definition of "recent” to three months allowed us to include a sufficient number of cases in the treatment group, ensuring the feasibility of the analysis.*

2. Please include reference about socioeconomic analysis, why do not use ABEP criteria or other standart SES criteria? please detail the household assets included

**Answer:** *The ABEP criterion was not used in this socioeconomic analysis because it considers the ownership of goods and services unsuitable for the selected municipalities' socioeconomic reality. Items such as dishwashers and domestic workers, for example, are uncommon in these areas and were therefore not included in the questionnaire. As a result, it would not have been possible to make meaningful comparisons using the ABEP criterion. For this reason, we opted to use the MJCA.* *To provide information on the household assets, we included boxplots for each item used to construct the socioeconomic indicator as supplementary material, and mentioned the supplementary material* ***on page 9****.*

*“To assess the socioeconomic status of the participant’s household, a socioeconomic index was estimated using Multiple Joint Correspondence Analysis (MCA) according to the presence of household assets. Details of the variables used in the analysis are available in the supplementary material (S1 Figure)”*

3. Line 184 please include reference for score interpretation.

**Answer:** *Suggestion accepted. We rewrote the sentence* ***on page 9*** *for better clarity.*

*“The socioeconomic index had a mean score of 49.5 on a scale from 0 to 100, indicating that the average socioeconomic status in the sample is near the midpoint of the scale, with a standard deviation of 12.5.”*

4. Line 208 - VAS instead of EAV.

**Answer:** *Correct. We changed EAV to VAS.*

5. Line 220- it is not clear how do you separate the effect of malaria with the previous poor conditions - residual counfounding.

**Answer:** *We used PSM to control for these factors and minimize the risk of residual confounding. In the matching process, we considered pre-existing morbidities, along with the socioeconomic and demographic conditions of the individuals. This approach allowed us to create control and treatment groups exposed to the same context and sharing similar epidemiological profiles. By doing so, we aim to isolate the specific effect of malaria, ensuring that any observed differences between the groups are not due to pre-existing health or socioeconomic disparities.*

6. Line 261 - reference 19 and 20 are incomplete.

**Answer:** *The references were corrected.*

7. Line 296 it is not prevention, please rewrite the sentence.

**Answer:** *Suggestion accepted. We rewrote the sentence on page 19.*

*“(...) However, there is evidence of low individual adherence to preventive measures, such as the use of long-lasting insecticidal nets. Additionally, individuals often interrupting treatment when symptoms improve [26-28]”.*

8. Line 297 I suggest to include the distance from healthcare units, sometimes days.

**Answer:** *Suggestion accepted. We included distance from healthcare units in the sentence* ***on page 19****.*

*“Adherence to antimalarial treatment is associated with socioeconomic conditions, access to healthcare (access to self-medication, distance to healthcare units, and monitoring of treatment by healthcare agents), knowledge of the disease, and drug administration - treatment duration, presence of symptoms and side effects) [29].”*

9. Line 300 adhence to treatment or prevention?

**Answer:** *Suggestion accepted. We rewrote the sentence* ***on page 19*** *to clarify that it refers to non-adherence to treatment.*

*“Empirical evidence for the Brazilian Amazon has shown that non-adherence to treatment varies from 30 to 40%, depending on the drug treatment and the method used to measure adherence [30].”*

10. Please exclude the sentence: "Continued investment in malaria control programs and improved access to health services are essential to mitigate the negative impact of this disease on the quality of life of affected populations". It is not supported by the data.

**Answer:** *Suggestion accepted. We removed the sentence* ***on page 20****.*
